# Trends in Ocean Colour and Chlorophyll Concentration from 1889 to 2000, Worldwide

**DOI:** 10.1371/journal.pone.0063766

**Published:** 2013-06-12

**Authors:** Marcel R. Wernand, Hendrik J. van der Woerd, Winfried W. C. Gieskes

**Affiliations:** 1 Royal Netherlands Institute for Sea Research, Physical Oceanography, Marine Optics & Remote Sensing, Texel, The Netherlands; 2 Institute for Environmental Studies (IVM), VU University Amsterdam, Amsterdam, The Netherlands; 3 University of Groningen, Department of Ocean Ecosystems, ESRIG, Groningen, The Netherlands; Universidade Federal do Rio de Janeiro, Brazil

## Abstract

Marine primary productivity is an important agent in the global cycling of carbon dioxide, a major ‘greenhouse gas’, and variations in the concentration of the ocean's phytoplankton biomass can therefore explain trends in the global carbon budget. Since the launch of satellite-mounted sensors globe-wide monitoring of chlorophyll, a phytoplankton biomass proxy, became feasible. Just as satellites, the Forel-Ule (*FU*) scale record (a hardly explored database of ocean colour) has covered all seas and oceans – but already since 1889. We provide evidence that changes of ocean surface chlorophyll can be reconstructed with confidence from this record. The EcoLight radiative transfer numerical model indicates that the *FU* index is closely related to chlorophyll concentrations in open ocean regions. The most complete *FU* record is that of the North Atlantic in terms of coverage over space and in time; this dataset has been used to test the validity of colour changes that can be translated to chlorophyll. The *FU* and *FU-*derived chlorophyll data were analysed for monotonously increasing or decreasing trends with the non-parametric Mann-Kendall test, a method to establish the presence of a consistent trend. Our analysis has not revealed a globe-wide trend of increase or decrease in chlorophyll concentration during the past century; ocean regions have apparently responded differentially to changes in meteorological, hydrological and biological conditions at the surface, including potential long-term trends related to global warming. Since 1889, chlorophyll concentrations have decreased in the Indian Ocean and in the Pacific; increased in the Atlantic Ocean, the Mediterranean, the Chinese Sea, and in the seas west and north-west of Japan. This suggests that explanations of chlorophyll changes over long periods should focus on hydrographical and biological characteristics typical of single ocean regions, not on those of ‘the’ ocean.

## Introduction

Oceanographic data archaeology (salvaging historical ocean data) is, according to Parker [1], a critical requirement for climate change research when the data (e.g., temperature and salinity) cover long time periods. Changing climate will affect marine life, and this, in turn, plays a crucial role in climate control [2]. As a matter of fact, phytoplankton accounts for nearly half of Earth's total primary productivity [3]. Phytoplankton biomass is nowadays readily derived from satellite data [4]; indeed, chlorophyll provides the best index of phytoplankton biomass for primary productivity studies, as was recently confirmed by Huot *et al*. [5]. Long-term changes in ocean primary production may well have important consequences for the ocean food chain, as well as for the global carbon cycle [6].

One of the so-called ‘Essential Climate Variables’ listed by the World Meteorological Organization (WMO) to detect biological activity in the ocean's surface layer is ocean colour [7]. More recently an important aspect of climate research has been the detection of ocean colour from space with derived products such as water transparency that is influenced by coloured dissolved organic matter absorption and chlorophyll concentration. Water transparency can be used to predict the depth of the upper ocean mixed layer [8]. This layer plays a critical role in the flux of energy between atmosphere and ocean.

Changes in water colour are caused by a change in the composition of optically active substances [9]: suspended particulate matter, pigments (chlorophyll-a, b and c and carotenoids) in algae [10], and dissolved organic matter [11]. The blue colour of oligotrophic oceans is caused by domination of selective absorption and scattering of the water molecules. Chlorophyll colours the water green (or even red by the carotenoids peridinin in dinoflagellates), and the presence of coloured dissolved organic matter (CDOM) will, in high concentrations, account for the absorption of most of the blue part of the incoming sunlight, resulting in brownish-coloured water.

The colour of seas and oceans is, next to water transparency, salinity and temperature, one of the few oceanographic parameters that have been recorded for over a century [12]. One outstanding long-term record (since 1931) has been produced by the Continuous Plankton Recorder Surveys in the North Sea and the North Atlantic [13], [14]; other records are the Hawaii- and Bermuda Atlantic Ocean Time-Series (HOTS and BATS [15], [16]) and the California Cooperative Oceanic Fisheries Investigations (CalCOFI) [17]. All these series have from the launch of the Coastal Zone Color Scanner been supplemented with satellite-derived oceanographic products for temperature change and, as to biological characteristics, for ocean colour and its derivative, chlorophyll concentration. From the late 1990s more sophisticated imaging ocean colour satellite sensors such as the Sea viewing Wide angle Field of view Sensor SeaWiFS, the MEdium Resolution Imaging Spectrometer MERIS and the Moderate Resolution Imaging Spectroradiometer MODIS sample the ocean's colour. Since the launch of SeaWiFS, ocean colour from space with rigid measurement protocols [18] has been archived as a standard oceanographic parameter. With revisit times of 1 to 3 days, a swath width of around 1,000 to 2,000 kilometres and a spatial resolution of 250 to 1,000 meters, the ocean has never before been sampled optically on such a scale [19].

The record of chlorophyll changes over time is limited; only a few authors have presented analyses of trends in chlorophyll over limited numbers of years. Venrick et al. [20] found, by means of trend analysis of standing-stock chlorophyll, a doubling of phytoplankton chlorophyll in the central North Pacific gyre between 1964 and 1985. We must bear in mind that the analysis of chlorophyll has become more accurate with the introduction of HPLC in the late seventies of the past century [21]. Falkowski and Wilson [22] found areas (10 by 10 degrees boxes) with significant positive and negative average annual changes in Secchi depth since 1900, but overall their estimated annual change averaged over the whole North-Pacific Ocean's chlorophyll concentration is small and not significant. A similar transformation was recently used by Boyce *et al*. [23] in order to derive world-wide changes in ocean phytoplankton biomass since 1899.

**Figure 1:**
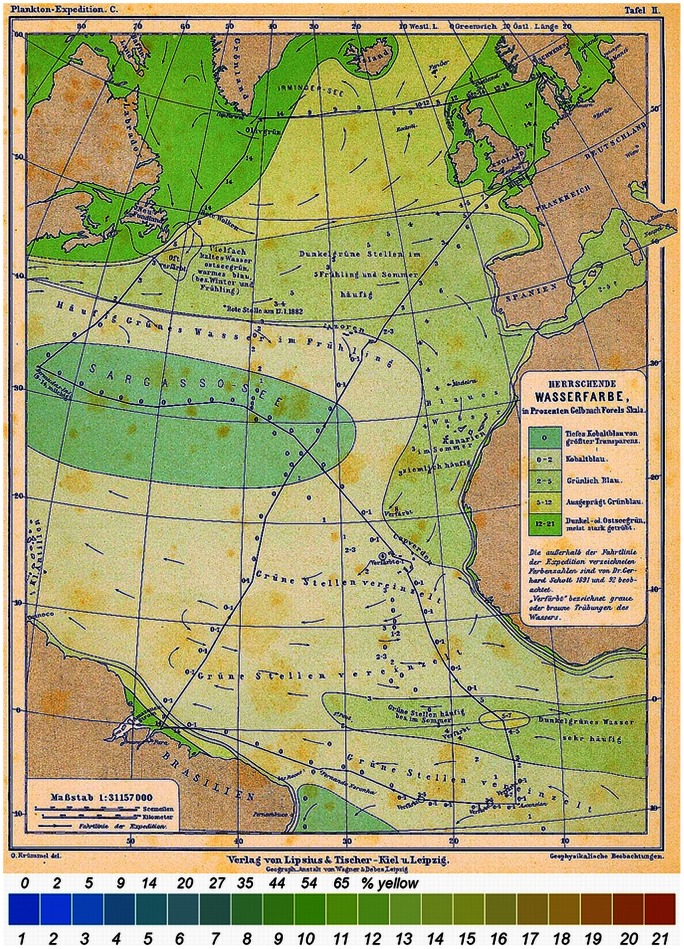
Krümmel's contoured North Atlantic *FU*-map (1889). The sailing track of the steamship ‘*National*’ is shown in black. The colour (Wasserfarbe) was indicated as a percentage of a yellow potassium chromate solution added to a blue copper-sulphate solution. The legend indicates the *FU*-scale colours 1 to 21.

Since 1889 a colour comparator method has been used to establish the colour of the sea, through Forel-Ule scale observations [24], [25]. Besides Forel and Ule, the inventors of the scale, Krümmel (1893), Luksch (1901) and Von Drygalski (1897) [26], [27], [28] were among the first to use this method in the open sea. Krümmel made his observations during the plankton expedition of the Humboldt Society in 1889. At that time only the blue to blue-green part of the scale (scale numbers 1 to 11) was used to classify the colour of open water. An example of Krümmel's contoured sea colour map is shown in Figure 1. This map shows surprisingly well the oligotrophic Sargasso Sea gyre (‘deep cobalt blue, *FU*-1’), as well as the outflow of the Congo River (‘greenish-blue, *FU*2-3’), and the Canary and Greenland Currents (‘dark green, *FU*4-7’). The data were collected in the Atlantic during the Plankton Expedition of 1889 and the colour of the sea was expressed as a percentage of a yellow potassium chromate solution added to a blue copper-sulphate solution. Luksch collected observations over eight years during the ‘*Pola*’ expedition (1890–1898) in the Mediterranean, Aegean- and Red Sea and Von Drygalski performed observations during the Greenland Expedition of the Geographical Society of 1891–1893. Since 1890 the Forel-Ule scale became the most commonly used and most simple scale to determine, through comparison, the colour of seas, lakes and rivers.

Over the years only a limited number of geographical maps, based on interpolation of sets of Forel-Ule data, became available. The U.S. Navy Hydrographic Office published in the 1950s three atlases on marine geography, including Forel-Ule contours of the Seas of Japan, Korea and Indochina [29], [30], [31]. A more comprehensive effort was made by Frederick [32], who presented contoured Forel-Ule maps. She had around 24,000 Forel-Ule observations at her disposal to create contour maps of averaged sea colours, but she did not analyse trends. Wernand and Van der Woerd [33] have recently found, in the North Pacific Ocean, a greening trend from 1930 until 1955, and since then a bluing, based on an analysis of 17,171 *FU*-observations that are available for that ocean.

The aim of the work presented here was to visualise changes in ocean colour, notably its chlorophyll component, over the longest possible period of time by an analysis of the ‘forgotten’ globe-wide ocean colour obtained by the Forel-Ule method. The temporal variation of ocean colour has been analysed from 1889 till the year 2000, based on subsets in a total of 221,110 *FU* observations collected globe-wide. First the data selection and quality control procedures are described and a model is introduced that provides simple relations for the conversion from *FU*-scale number to Chlorophyll concentration. Subsequently, the data are grouped in 28 seas and oceans. All this should eventually allow speculation on mechanisms behind basin-scale external forcing of the plankton abundance near the ocean surface.

## Materials and Methods

With the Forel-Ule method the colour of natural waters is compared to a scale, that consists of twenty-one tubes filled with mixtures of coloured chemical solutions. The method has recently been described by Wernand and van der Woerd [34]. Most *FU* observations were retrieved from oceanographic and meteorological databases archived by NOAA-NODC [35]. This dataset contains, besides date, geographical position and Secchi depth (*SD*), the *FU* index (coded 1 to 21; see introduction). Two new attributes were added for each entry, indicating the meteorological season and the sea area. The geographical naming follows the conventions of the U.S. Geological Survey (USGS) that is based upon the latitude and longitude at which the observation took place. This dataset contains 220,440 observations collected from 1907 to 1999, inclusive.

From three historic expeditions, between 1889 and 1899, 670 observations were digitized and added to the NOAA-NODC dataset. The added *FU*-observations from before 1900 consisted of 1) 89 observations from Krümmel's plankton expedition in the North Atlantic in 1889, 2) 367 observations from Luksch ‘*Pola*’ expeditions collected from 1890 to 1898 in the Mediterranean, Aegean- and Red Sea and 3) 214 observations from Schott's German deep sea expedition on the steamer ‘*Valdivia*’ collected from 1898 to 1899 in the North Sea, Atlantic, Indian Ocean, Red Sea and Mediterranean. During Von Drygalski's Greenland Expedition of the Geographical Society of 1891–1893 around 70 *FU* observations were collected in the North Sea, North Atlantic, Davis Strait and Baffin Bay; however the bad quality of the printed map made it impossible to digitize these data. For most of the *FU* data a Secchi disc depth observation was also available. For the first quality check we looked at the combination of *FU* and *SD* values and omitted data which seemed incorrect, the criterion being that low *FU* values should have high *SD* values and *vice versa*.

**Figure 2:**
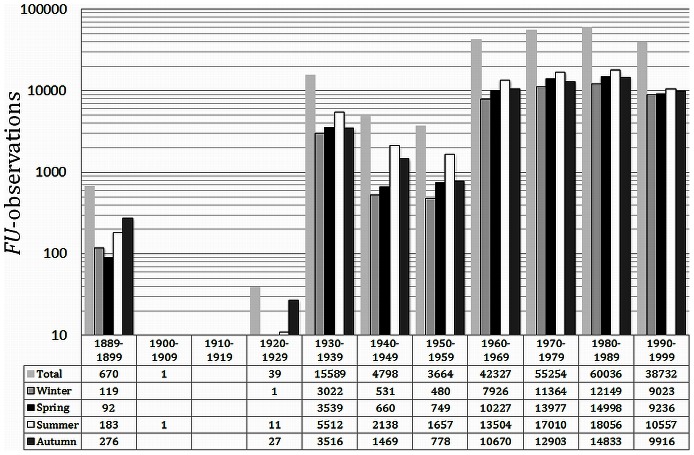
The temporal distribution of the total number of 221110 *FU*-observations contained within dataset-0 for the period 1889 to 1999. Data is binned per season and per decade except for the first period of 11 years from 1889–99. Notice: the period 1900 to 1929 contain zero or a limited number of observations.

The re-formatted dataset is referred to as dataset-0 and contains, after the first quality check, 221,110 *FU* globally collected observations of open water, lakes and rivers. Figure 2 shows a bar chart of the total number of *FU* observations collected within a decade and per season. The period from 1889 to 1899 period covers eleven years in order to gain as much available information of the earliest period of ocean colour observation.

### Data filtering

Subsequently, dataset-0 was differentiated into three geographical areas by applying Basin Masks (BM). The Basin Masks are used to filter out observations within land boundaries (lakes and rivers), and observations close (100 km or 500 km) to the coast. The Basin Masks were created and executed in ArcGIS 9.3 software [36] by means of the ArcGIS clip function.

First, BM1 was created to extract data from dataset-0 free of observations within the land boundaries just mentioned. This dataset is referred to as dataset-1 and contains 219,839 sea observations. Table 1 shows the distribution of the *FU* observations per sea area with details on the total and seasonal numbers of *FU* observations, also expressed as a percentage of the total. The definition of seasons follows the meteorological convention. We highlight some results of the major oceans and seas. The selected oceans and seas are the Barents Sea, Bering Sea, East China Sea, Indian Ocean, Mediterranean, North- and South Atlantic, North- and South Pacific, Norwegian Sea, Pacific Coast, Philippine Sea, South China Sea, Sea of Japan, Sea of Okhotsk and the Yellow Sea.

**Table 1:** The distribution of *FU*-observations contained within dataset-1.

Sea Area	Abr.	Total	W	W-%	Sp	Sp-%	S	S-%	A	A- %
Antarctica	AA	51	48	94.1	3	5.9	0	0	0	0
***Atlantic Ocean***	***AT***	**8161**	1318	16.2	1823	22.3	3090	37.9	1930	23.7
Arctic Ocean	ARO	1	0	0	0	0	1	100	0	0
Baltic	BAL	122	0	0	47	38.5	58	47.5	17	13.9
***Barents Sea***	***BAR***	**2651**	25	0.9	477	18.0	1741	65.7	408	15.4
Baffin Bay	BFB	5	0	0	0	0	4	80	1	20
Black Sea	BL	110	1	0.9	31	28.2	28	25.5	50	45.5
Bay of Biscay	BOB	17	2	11.8	3	17.6	7	41.2	5	29.4
***Bering Sea***	***BS***	**1750**	14	0.8	56	3.2	1645	94.0	35	2
Caribbean Sea	CB	700	179	25.6	158	22.6	210	30.0	153	21.9
Chukchi Sea	CS	50	0	0		0	50	100	0	0
***East China Sea***	***ECS***	**3432**	638	18.6	859	25.0	1122	32.7	813	23.7
Gulf of Alaska	GAK	279	1	0.4	75	26.9	179	64.2	24	9
Gulf of Mexico	GM	110	3	2.7	28	25.5	27	24.5	52	47.3
Greenland Sea	GRS	21	0	0	4	19.0	13	61.9	4	19.0
***Indian Ocean***	***INO***	**3446**	971	28.2	711	20.6	552	16.0	1212	35.2
Kara Sea	KRA	97	0	0	0	0	53	54.6	44	45.4
***Mediterranean***	***MED***	**1080**	163	15.1	155	14.4	366	33.9	396	36.7
North Sea	NS	56	4	7.1	9	16.1	22	39.3	21	37.5
***Norwegian Sea***	***NWS***	**1524**	27	1.8	277	18.2	1121	73.6	99	6.5
***Pacific Coast***	***PC***	**1842**	303	16.4	461	25.0	522	28.3	556	30.2
***Pacific Ocean***	***PAC***	**34611**	6281	18.1	7417	21.4	11077	32.0	9836	28.4
***Philippine Sea***	***PHS***	**106557**	24931	23.4	27115	25.4	27873	26.2	26638	25.0
Red Sea	RS	166	51	30.7	61	36.7	0	0	54	32.5
***South China Sea***	***SCS***	**3055**	760	24.9	889	29.1	813	26.6	593	19.4
***Sea of Japan***	***SJP***	**39198**	6432	16.4	10399	26.5	13371	34.1	8996	23
***Sea of Okhotsk***	***SOK***	**1393**	18	1.3	261	18.7	897	64.4	217	15.6
***Yellow Sea***	***YS***	**9354**	2244	24	1952	20.9	3323	35.5	1835	19.6
**Total**		**219839**	44414	20.2	53271	24.2	68165	31	53989	24.6

The table shows from left to right: the seas for which *FU* data was collected, its abbreviation, the total number of observations, the observations per season (Winter (W), Spring (Sp), Summer (S) and Autumn (A)) with the percentage of the total number of observations. Seas discussed in this article are in bold italic. Observations of the inland Caspian Sea (465 obs.) are filtered out using BM1.

Second, BM2 was chosen pragmatically to extract open sea observations from dataset-0, at a distance of more than 100 km off-coast, to avoid effects on water colouration of anthropogenic pressure in the coastal zones such as locally increased nutrient loading (eutrophication) that tends to enhance phytoplankton biomass or high sediment loading caused by changes in land use or erosion. These phenomena result in short-term, drastic colour changes, no doubt influencing the open sea *FU* values. This dataset is referred to as dataset-2 and contains 61.434 open sea observations. The last mask, BM3, was again chosen pragmatically to extract observations from dataset-0 at a distance of over 500 km from the coast to include the oceans but at the same time avoid the influences of mixing with the differently coloured water of nearby seas. This dataset will be referred to as dataset-3 and contains 21.971 open ocean observations.

**Figure 3:**
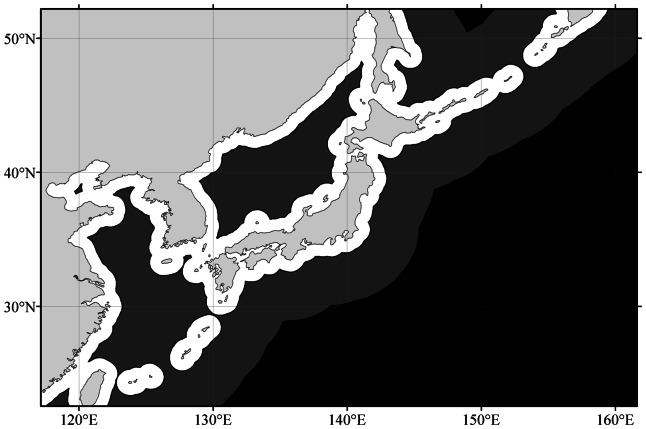
Example (around Japan) of three defined masks to extract *FU*-observations; in white the BM1 mask starting at the land/sea boundary spreading over the whole sea with on top of this layer, in dark grey, BM2 starting at a distances of >100 km off land and on top of this layer BM3, a layer in black, starting at a distance of 500 km off land.

An example of the shapes of the three data extraction masks is given in Figure 3. One must bear in mind that by clipping the data to the dimensions of the mentioned masks the number of observations per sea area diminish by roughly a factor of 4 in case of BM2 and a factor 10 in case of BM3. However, to establish a realistic colour of the bulk water of a sea or ocean, not influenced by its nearby coastal or surrounding seawater (with much higher *FU* numbers) this action is unavoidable. The data extraction masks, datasets name and the number of observations included in the dataset are summarized in Table 2. From dataset-1, a global map of the *FU*-colour distribution was established through an Inverse Distance Weighted interpolation (IDW) [37], [38]. IDW interpolation explicitly implements the assumption that observations that are close to one another are more alike than those that are further apart. The IDW determines cell values by calculating a linearly weighted combination of nearby *FU*-observations. The weight is calculated by taking the inverse of the distance between the cell and *FU*-observation, raised to a power (usually equal to two). This power option controls the significance of known points on the interpolated values, based on their distance from the output point. Datasets-2 and -3 are statistically analysed per sea area and per year. Data were binned per year to eliminate any influence of a seasonal cycle in the colour of the sea. The arithmetic mean *FU* values 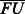 with the confidence interval of the mean (95%) and of the observations (95%) [39] of each sea were calculated. Linear trends over time were calculated by a, to the number of observations, weighted- linear regression of the (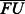) values. The weighted linear regression takes into account the number of observations as weights [40].

**Table 2:** The number of *FU*-observations per dataset unmasked and obtained by the use of 3 data-extraction masks.

Mask	-	BM1	BM2	BM3
***FU*** **-Dataset**	Dataset-0	Dataset-1	Dataset-2	Dataset-3
**Off-Coast**	+ lakes/rivers	0 km	>100 km	>500km
**No. of Obs.**	221110	219839	61434	21971

Off-coast indicates the areas where *FU*-data were collected.

To check the presence of a monotonously increasing or decreasing trend in the observations the nonparametric Mann-Kendall trend test per sea and ocean has been applied as proposed by Mann [41] and further studied by Kendall [42] and Sen [43]. The test was found to be a good tool for trend detection and has been utilized by many other investigators in the analysis of various types of environmental data [44], [45], [46]. For the statistical analyses including the Mann-Kendall test we applied the Excel add-in XLSTAT-software (http://www.xlstat.com). In the trend analysis all results are presented under the assumption that the *FU*-measurements have been made independently. The potential influence of temporal and spatial autocorrelation in the dataset on the simple linear regression analysis has not been taken into account, mainly because this is a very complex and certainly not well defined exercise [47]. Therefore the provided temporal trends should be treated with caution; we are aware of the fact that they provide a simplified description of reality.

### Bio-optical Modelling

An *FU* colour change from a lower to a higher scale index indicates a greening of seawater caused by the presence of light-absorbing substances, notably chlorophyll and CDOM. In order to simulate the relation of chlorophyll or CDOM and the *FU* index the HydroLight-EcoLight [48] modelling software was adopted. This model solves the radiation–transfer equations of light in natural waters and computes radiance or irradiance distributions and derived quantities, like remote sensing reflectance *R*_RS_ and *FU* [49]. By varying the model's input parameters, like chlorophyll, CDOM or mineral concentrations, a relation can be established between concentrations and *R*_RS_ or the *FU* index. HydroLight-EcoLight employs mathematically sophisticated invariant imbedding techniques to solve the radiative transfer equation. Details of this solution method can be found in Mobley's book on *Light and Water* [50] and in his ‘Comparison of numerical models for the computation of underwater light fields’ [51]. Both models, HydroLight and EcoLight, generate the same output; notice the difference, namely that HydroLight computes the full 3D-radiance distribution, whereas EcoLight computes only irradiances.

Two simulation runs were made to establish the influence of either chlorophyll or CDOM on the colour of seawater i.e. on the remote sensing reflectance *R*_RS_ and on the *FU* number. For modelling the oceanic waters, the new IOP model “NEW” case 1 was used. This model is based on a combination of i) the results of modelled particle absorption as given by Bricaud *et al.* [52] and ii) the results of modelled particle absorption described in more recent publications by Morrison and Nelson [53] and Vasilkov *et al*. [54]. The “NEW” case 1 two-component IOP model consists of 1) pure water [55] and 2) chlorophyll-bearing particles with co-varying CDOM and detritus. One of the model's output parameters is the remote sensing reflectance *R*_RS_(λ) which was automatically convoluted with CIE1931 curves into a chromaticity coordinate set *x, y* and accordingly into a *FU* scale number (see [34] for more details). In this way a relation between chlorophyll and *FU* could be established for oceanic waters alone, and is used in the trend analysis to convert *FU* back into a chlorophyll concentration.

For seas we used a generic four-component case 2 water specifying concentration profiles and IOP models for each of four components: 1) pure water [55], 2) chlorophyll bearing particles (*a*_p_*(λ) and *b*(λ)  = 0.4070^0.7950^ * (660/λ)^1^), 3) CDOM given as absorption in m^−1^ at 440 nm not co-varying with chlorophyll bearing particles (a_CDOM_*(z, λ)  = 1 exp [−.0140 (λ−440)]), and 4) mineral particles (*a*_p_*(λ) and *b*_p_*(λ) of calcareous sand). For the phase function we used Morel *et al*. small particle Case 1 phase function [56].

To simulate the chlorophyll and mineral concentration in open sea water the values of these properties were set respectively at 0.1 mg m^−3^ and 0 or 0.2 g m^−3^. The CDOM absorption (a_440_) was varied between 0.01 and 1.0 m^−1^. Again, the calculated remote sensing reflectance *R*_RS_(λ) was convoluted with CIE1931 curves into a chromaticity coordinate set *x, y* and into a *FU* scale number [34]. In Table 3 the EcoLight model parameters, including the scattering and absorption properties of the constituents are tabulated for both models.

**Table 3:** Specifications of the four components used in the Ecolight model for concentration ranges, inherent optical properties and atmosphere.

Ecolight IOP model specification	Pure water	Chlorophyll (mg m^−3^)	CDOM (m^−1^)	Minerals (g m^−3^)
**Case 1 – ocean chlorophyll dominated**	Pope & Fry	0.1 to 40	Co-varying with chlorophyll	-
**Case 2 – ocean CDOM dominated**	Pope & Fry	Constant with depth 0.1 mg m^−3^	Constant with depth varying from 0 to 1 m^−1^	Constant with depth 0 or 0.2 g m^−3^
**Specific absorption**	-	*a*_p_*(z, λ)	*a*_CDOM_*(z, λ)	*a*_p_*(λ) for calcareous sand
**Specific scattering**	-	*b*(z, λ)	-	*b*_p_*(λ) for calcareous sand
**Phase function**	-	Morel *et al*. [56]	-	Morel *et al*. [56]
** Sky model**	Normalised radiance by RADTRANX [57] (Harrison and Coombes, 1988)			
	Diffuse and direct sky by RADTRANX [58] (Kasten and Czeplak, 1980)			
**Parameters**	Cloud Fraction = 0, Solar Zenith = 50^0^, Press = 29.9 in. merc, Day of Year = mean earth-sun distance used, Air Mass Type = 1, Rel. Hum. = 80%, Wind Speed = 5ms^−1^, Visibly = 15km, Total Ozone = 300 Dobson units, Aerosol Opt. Thickness = 0.261			

## Results

**Figure 4:**
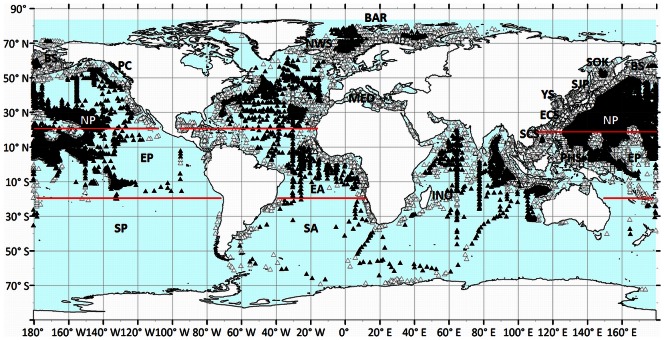
The positions of *FU*-observations extracted at a distance >500 km off-coast and at a distance of >100 km off-coast (resp.21971 obs.▴, 61434 obs.▵). Oceans and seas are indicated by abbreviations as mentioned in Table 2. Red lines indicate the division of north and equatorial regions. Although the Mediterranean has a limited number of 237 observations, it was found to be important to analyse this sea for which data were already collected at the end of the 19^th^ century.

**Figure 5:**
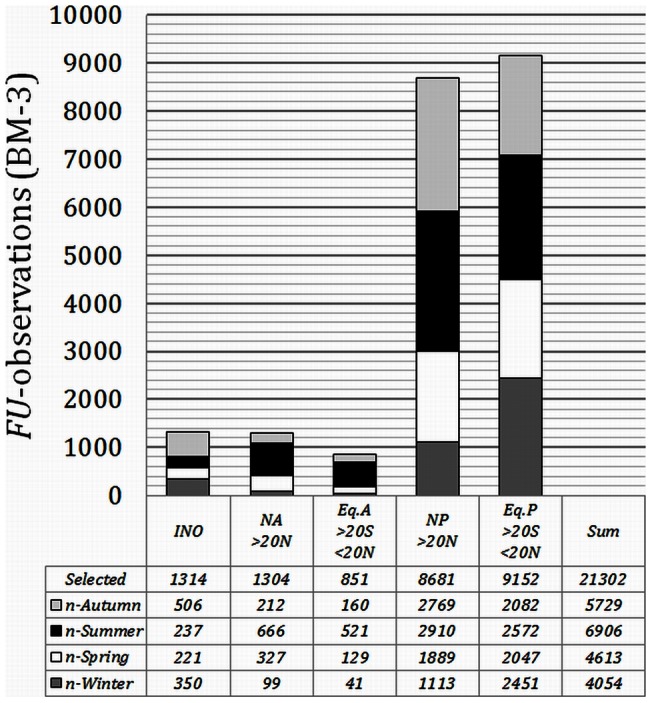
The selected 21302 *FU*-observations extracted under the ocean mask BM3, binned per sea area and per season.

**Figure 6:**
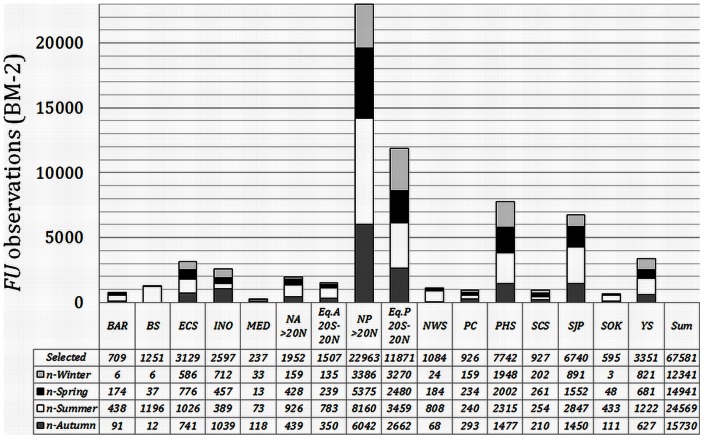
The selected 67581 *FU*-observations extracted under the basin mask BM2, binned per sea area and per season.

Figure 4 shows the geographical positions of the observations extracted under respectively BM3 (>500 km, black triangles) and BM2 (>100 km, grey-white triangles). Parts of the globe that are under-sampled are mainly found in the Southern Hemisphere. For further analysis the Atlantic and Pacific were split into a northern (NA, NP) and an equatorial (EA, EP) part, indicated in both figures by red lines. The southern parts of both oceans were not analysed; for the South Atlantic no data were available for the period 1900 to 1959 and the remaining periods were highly under-sampled; and as to the South Pacific observations are limited to the period 1950 to 1999. However, during this period, only the last two decades have been sampled properly. The Indian Ocean has been analysed as one single entity. The selected and analysed seas are the Barents Sea, Bering Sea, East China Sea, Mediterranean, Norwegian Sea, Pacific Coast, Philippine Sea, South China Sea, Sea of Japan, Sea of Okhotsk, and Yellow Sea. Figure 5 shows the number of available *FU* observations of the selected oceans extracted under BM3 (>500 km off-coast). Because the statistical analysis was also done for each separate season, the number of seasonally binned observations is given too. In Figure 6 these numbers are provided for all seas that were treated under BM2 (>100 km off-coast).

In order to understand the oceanic trend analysis of chlorophyll, we first present the Ecolight modelling results of the conversion between *FU* and chlorophyll concentration (chapter 3.1).

Hereafter, a global view of averaged ocean colour of the oceans and seas utilizing all observations from 1889 to 2000 is presented in chapter 3.2. The chapter is followed by a regional trend analysis, first per ocean and then per sea.

### *FU* Modelling

**Figure 7:**
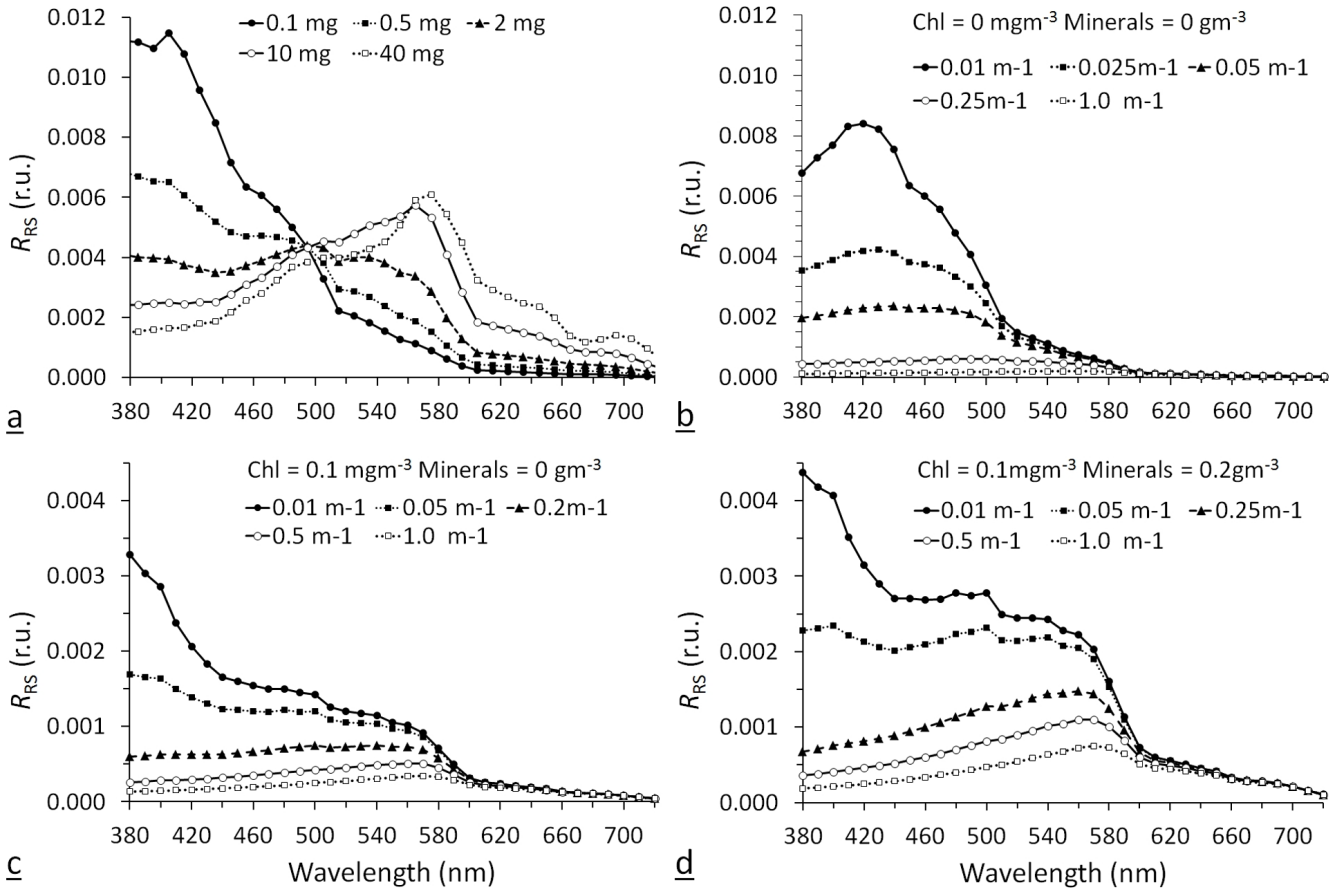
Examples of the Ecolight modelled *R*_RS_ (sr^−1^) for case 1 waters (panel a) and case 2 waters (panels b, c and d) with variable composition (see also **Table 4**). In panel (a) only the input chlorophyll concentration is varied between 0.1 and 40 mg m^−3^. For case 2 waters the input CDOM_440_ absorption (a_440_) is varied between 0.01 and 1.00 m^−1^ with chlorophyll and mineral concentration of both 0 (panel b), fixed chlorophyll concentration of 0.1 mg m^−3^ and a mineral concentration of 0 (panel c) and fixed chlorophyll concentration of 0.1 mg m^−3^ and a mineral concentration of 0.2 g m^−3^ (panel d). From these *R*_RS_ spectral signatures the chromaticity coordinate set and *FU* number were calculated.

**Figure 8:**
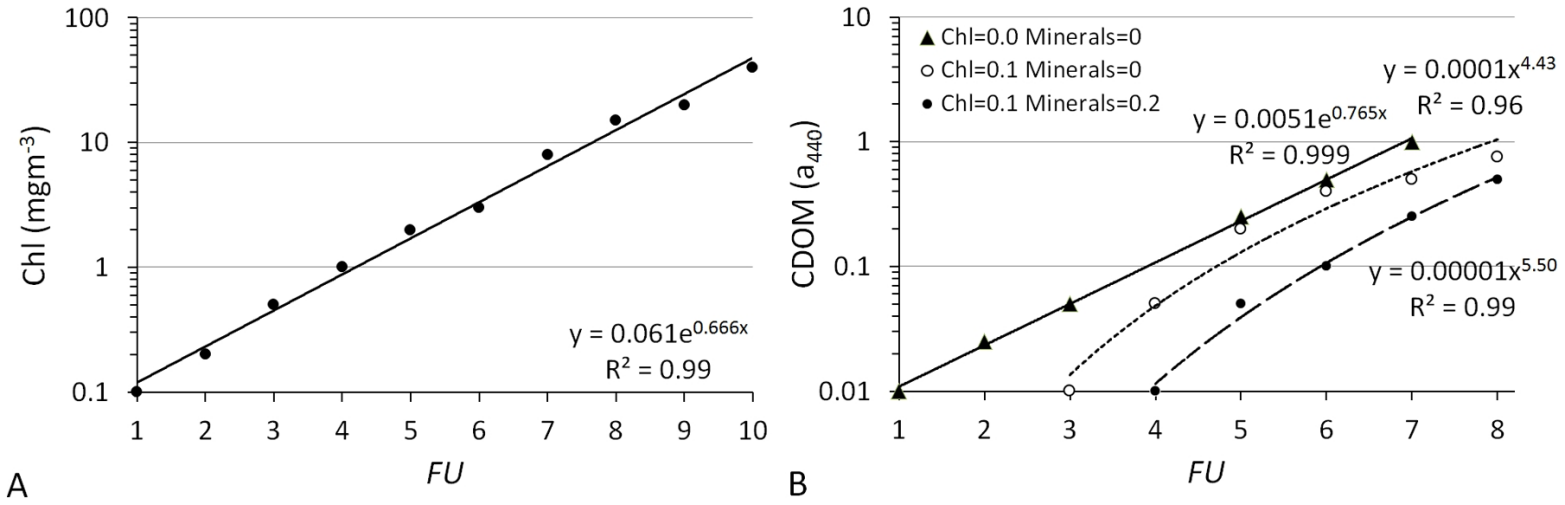
Chlorophyll concentration and CDOM attenuation at 440 nm as a function of *FU*-number. The *FU*-numbers were derived from the Ecolight *R*_RS_ spectra. In the left panel (A), chlorophyll was varied from 0.1 to 40 mg m^−3^ with a resulting span of *FU*1 to *FU*10. For case 2 waters (panel B), the relationship between CDOM and *FU* was calculated for three combinations of chlorophyll concentration (0 or 0.1 mg m^−3^) and mineral concentration (0 or 0.2 g m^−3^).

Varying chlorophyll concentrations were used as input for the two-component case 1 water model, for the four-component case 2 water model CDOM as input was varied (a_440_ between 0 and 1 m^−1^) with fixed values for components three and four mimicking open sea bio-optical values. For each run the EcoLight modelling software output parameters are the spectral remote sensing reflectance *R*_RS_(λ), as shown in Figure 7, and the *FU* number shown in Figure 8.

Figure 7a shows *R*_RS_ spectra generated by the case 1 model with varying chlorophyll, Figure 7b shows *R*_RS_ spectra generated by the case 2 model with varying CDOM absorption and chlorophyll and mineral concentrations set to 0. Figures 7c and 7d show *R*_RS_ spectra generated by the case 2 model with varying CDOM and chlorophyll set to 0.1 mg m^−3^ and minerals set to 0 and to 0.2 g m^−3^ respectively.

The results presented in Figure 8A show a change in chlorophyll concentration from 0.1 to 1 mg m^−3^ resulting in a shift in *FU* index from 1 to 4. An exponential fit to the model results provides the following relation:
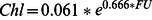
(1)


This relation takes into account the fact that in the open ocean chlorophyll bearing particles are co-varying with CDOM and detritus, whereas detritus contributes only 5% to 20% to the non-water absorption coefficient [59]. Equation 1 is used to transform the acquired 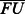 into an average chlorophyll concentration per year for oceanic regions. After having consulted a chlorophyll composite of an entire MODIS mission, with maximum chlorophyll concentration of a few mg m^−3^, we decided to set the upper limit of observed *FU* to 7 (≈6 mg m^−3^ chlorophyll) to avoid extreme outliers which could greatly influence the results; remember the exponential relation between *FU* and chlorophyll (Equation 1). In this way, observations taken above extremely dense phytoplankton blooms (e.g., coccolithophorids) could be avoided. As an example (BM2), for the Indian Ocean no data were found >*FU*7, for the North Atlantic 25 observations were omitted and for the North Pacific (total of around 23,000 obs.) we omitted 240 observations for our chlorophyll calculations.

For the seas, results will be presented as 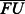 only, because they generally have more optically active constituents and no single relation between *FU* and chlorophyll can be established. However, to demonstrate the influence of CDOM, on the *FU* scale index we present case 2 model results for 3 modelled parameter settings. The parameter settings are identical to the settings used to generate the results in Figures 7b, 7c and 7d. In Figure 8B we see that a change in CDOM absorption from 0.01 to 0.1 m^−1^ results in a shift over the first 4 *FU* scale colours in the case that CDOM, next to water itself, is the only absorber of light. Through an exponential fit CDOM_440_ absorption can be calculated with a coefficient of determination *R* ^2^ = 0.999 according to
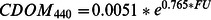
(2)


Setting both the background chlorophyll concentration to 0.1 mg m^−3^ and the mineral concentration to zero the *FU* numbers change from *FU* = 3 to *FU*≈8 (open circles in Figure 8B), which implies that the low chlorophyll concentration (set at 0.1 mg m^−3^) already colours the water to a *FU* value of 3. This outcome is somewhat different from the case 1 modelling results, shown in Figure 8A, where the same chlorophyll concentration corresponds to *FU* = 1. We must bear in mind that in the case 1 model, different from the case 2 model, CDOM is co-varying with chlorophyll. Through a power law fit CDOM_440_ absorption can be calculated with a coefficient of determination *R* ^2^ = 0.96 according to
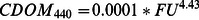
(3)


In the last set of results of the case 2 Ecolight model, the background chlorophyll concentration was set to 0.1 mg m^−3^ and the mineral concentration was set to 0.2 g m^−3^. For *FU* numbers between 4 and 8 the CDOM_440_ absorption can be calculated with a coefficient of determination *R* ^2^ = 0.99 according to
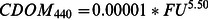
(4)


The CDOM-*FU* relations above are presented to help with the interpretation and comparison of a derived 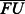 in seas for which more background knowledge of the area-specific inherent optical properties are available.

**Figure 9:**
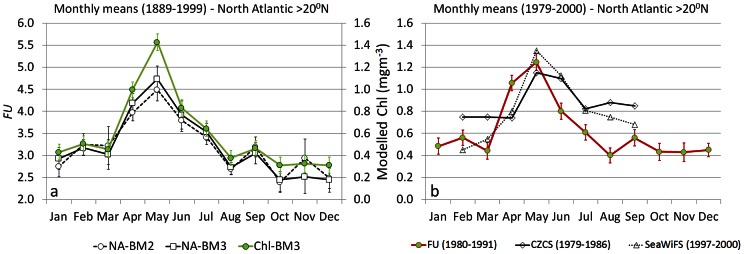
Representation of North Atlantic monthly 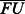 and chlorophyll for the periods 1889–1999 (a) and 1980–1990 (b, only chlorophyll). a – The BM2 (circles) and BM3 (squares) extracted *FU* data with error bars show the same pattern and reveal the North Atlantic spring bloom. On the secondary axes, indicated with green line/circles, the modelled chlorophyll (see Eq. 1) with error bars is shown. b – The modelled chlorophyll for the period 1980–1991, compared to CZCS- (1979–1988) and SeaWiFS (1997–2000) derived chlorophyll, blended with in-situ data, as presented by Gregg and Conkright, 2002).

In the following we show the significance i.e. accuracy and applicability of the *FU* scale observations presented here by visualizing i) an overall (1889–1999) monthly 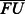 and by ii) visualizing an overall monthly chlorophyll concentration applying Equation 1. A representation of North Atlantic monthly means of *FU* and chlorophyll, including a comparison of satellite derived chlorophyll, are shown in Figures 9a and b. North Atlantic data, collected between 1899 and 1999, were extracted under the BM3 and BM2 mask to reveal possible divergence between both datasets, as the latter could be affected by coastal phenomena influencing the colour of the sea. The number of observations extracted under BM2 accounts for twice the number extracted under BM3. The data were converted to chlorophyll by Equation 1 and the monthly 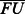 and chlorophyll were calculated. Similar results were found for both the analysed datasets, with an identified *FU* maximum (spring bloom) in May (Figure 9a). Calculated chlorophyll (BM3) shows a concentration of around 1.4 mg m^−3^ in May. Also a comparison was made between the satellite-based and *FU*-based chlorophyll seasonal cycle for the years 1980–1989. Figure 9b shows seasonal variations of the modelled chlorophyll concentration for the period 1980–1989 and the variation based on CZCS- and SeaWiFS-derived chlorophyll concentrations, blended with *in situ* data. The agreement, also (Figure 9a) with North Atlantic bloom values [60], is striking.

From the findings presented above we conclude that *FU* is a good proxy for chlorophyll in the open ocean. We have already argued that besides water itself chlorophyll (mostly inside the cells of phytoplankton) gives the ocean its colour [9]; but we must bear in mind that next to chlorophyll the usually non-covarying CDOM can, through absorption, have a similar influence on the colour of the sea and therefore on *FU*.

### Century averaged ocean colour

**Figure 10:**
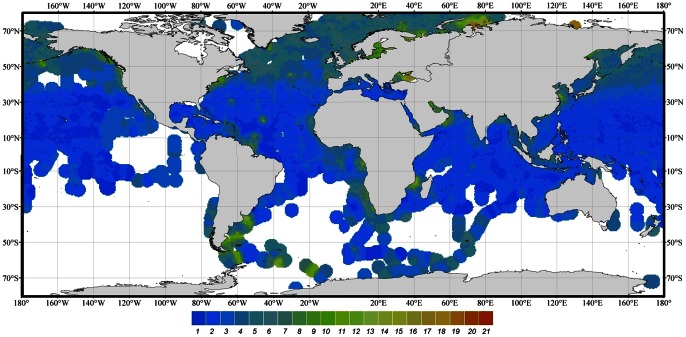
A global IDW interpolation of BM1 clipped *FU* observations. The weight is set to a power of 2. The search radius is set to 4 degrees with an output grid size of 0.5 degrees. Data collection period covers 1889 to 1999.

To establish a global view of the *FU* data collected between 1889 and 1999 all observations contained within dataset-1 were interpolated according to the IDW technique as described in material and methods. The inverse distance weight function was set to a power law of index 2. The search radius, that limits the number of *FU* observations used for calculating each interpolated *FU* value, was set to respectively 2.5 and 4 degrees with an output grid size of 0.5 degrees. The difference between results of interpolation using a search radius of 2.5 and 4 degrees turned out to be negligible, although for under-sampled seas the applied interpolation technique results in *FU* values for areas where no observation were performed. A map of IDW interpolated *FU* values, with a search radius of 4 degrees, is presented in Figure 10. The southern oceans, highly under-sampled, are shown in white.

A first analysis of Figure 10 shows that the lowest *FU* numbers are found in the Equatorial Pacific and the Indian Ocean (*FU*<2) and in the Equatorial Atlantic (*FU*<3). Note the extremely high *FU* values (16 to 21) east of the Ob and Yenisei estuaries in the Kara Sea (75°N, 70°E), most likely caused by very high CDOM values [61]. Also in the Baltic, where CDOM is the major light absorber [62], *FU* numbers of around 14 are encountered. The Atlantic shows increasing *FU* values (up to *FU* = 5) towards higher latitudes, most likely caused by the influence of more extensive phytoplankton blooms.

**Figure 11:**
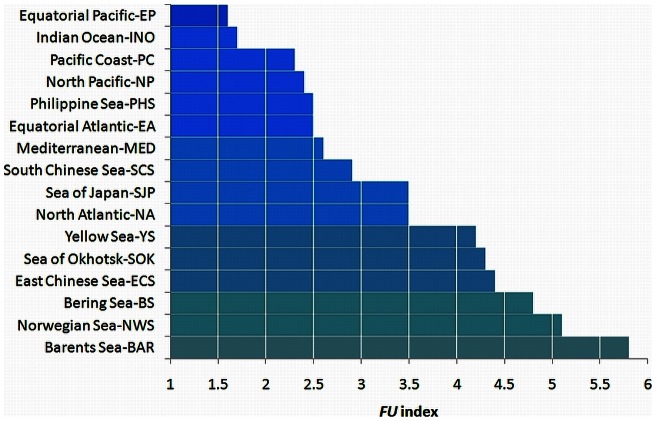
Representation of the oceans and seas identified and arranged in terms of their 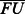
colour as calculated from all available observations extracted under BM2 or BM3 (see **Table 4** **).** The Barents Sea is the most greenish sea (bottom) and the Equatorial Pacific is the most bluish ocean (top).

Of all data extracted under either BM3 or BM2, the mean 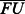
_1889–1999_ per sea area was calculated, with 95% confidence intervals for the mean and for the observations. The results of the statistical analysis show that the Equatorial Pacific and Indian Ocean are the bluest oceans of our globe with a mean 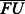 of 1.6 and 1.7, respectively. With the results of our statistical analysis one can classify and rank the oceans and greater seas in terms of their mean 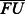 colour, presented in Figure 11 (similar to the IDW interpolation of Figure 10, however, less visible in this coloured graph). This figure shows that in the 20^th^ century the North Atlantic Ocean has been the greenest ocean of our planet while the Barents Sea was the greenest open sea of all.

**Figure 12:**
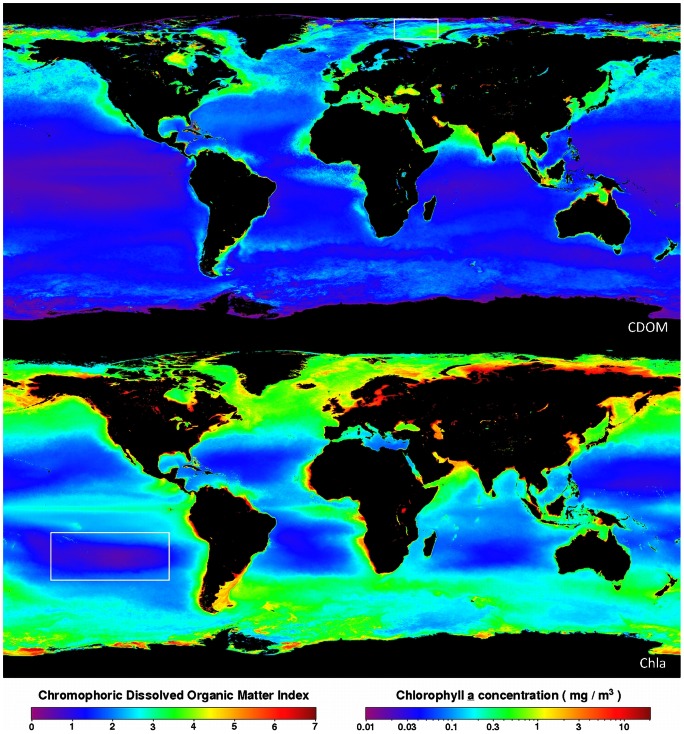
An entire MODIS mission composite map of the CDOM Index and chlorophyll-a concentration (4 July 2002–30 Jun 2010). The map shows a CDOM index <0.5 with the lowest oceanic chlorophyll concentrations of ≈ 0.02 mg m^−3^ in the South Pacific (white box left). The small white box at the top indicates the open water of the Barents Sea with a CDOM index ≈3.5 and chlorophyll concentrations ≈ 5 mg m^−3^. Source: level 3 browse, http://oceancolor.gsfc.nasa.gov/.

In order to support these classifications with recently collected global data (after 1999), we show in Figure 12 an entire MODIS mission composite (4 July 2002 to 30 Jun 2010) for the CDOM index [63] and for the chlorophyll-*a* concentrations. Note that in case 1 waters a mean relationship exists between the CDOM content and the chlorophyll concentration, anomalies in this relation, for both case 1 and case 2 waters are given by this so-called CDOM index Φ [63]. In the hyper-oligotrophic Equatorial Pacific gyre [64], located within the greater white box, we see a Φ<1 and very low chlorophyll concentrations, that are expected in blue oligotrophic oceans (around 0.02 mg m^−3^). Within the smaller white box, indicating the Barents Sea, we see a CDOM index around 3.5 and chlorophyll values around 5 mg m^−3^ colouring the sea green. Areas with an increasing CDOM index and or increasing chlorophyll concentration (Figure 12) are generally compatible with an increasing 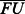
index (Figure 11).

### Yearly averaged regional chlorophyll and ocean colour

To establish the influence of the choice of Basin Mask on the trend analysis for open ocean water, we compared the results of dataset-2 (BM2) and dataset-3 (BM3). Because the conversion from *FU* index to chlorophyll concentration depends on the CDOM absorption, only oceanic waters with a CDOM index Φ<2.5 were converted with Equation 1; see also the global CDOM composite in Figure 12. For the remaining seas, as indicated in bold in Table 1, we applied the trend analyses on BM2 extracted data. For all data presented here, per sea or per ocean, the overall trend covers the whole period of data collection. The least-squares regression lines, indicated by a full blue or green line, indicate either a bluing or greening of the ocean/sea under investigation. For the oceans bluing/greening means decrease/increase in chlorophyll, for the rest of the seas this can mean a decrease/increase in 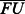 which can either mean a decrease/increase in chlorophyll or CDOM or a combination of both.

In the next paragraphs the results of our statistical analysis are presented per year per sea area for illustrational purposes only. For the Mann-Kendall test all single observations were used (no binning) In some cases, the BM2 extracted data is enclosed within the boundary of a neighbouring sea or ocean, hence a second data selection was needed, done with the ArcGIS data selection tool, to exclude overlapping data. The total number of extracted *FU* observations per sea or ocean can therefore be slightly different from the number of observations given in Table 2 and Figures 5 and 6. Data extracted under the BM3 mask concerns only the oceans, data extracted under the BM2 mask concerns both oceans and world seas.

#### Oceans

**Figure 13:**
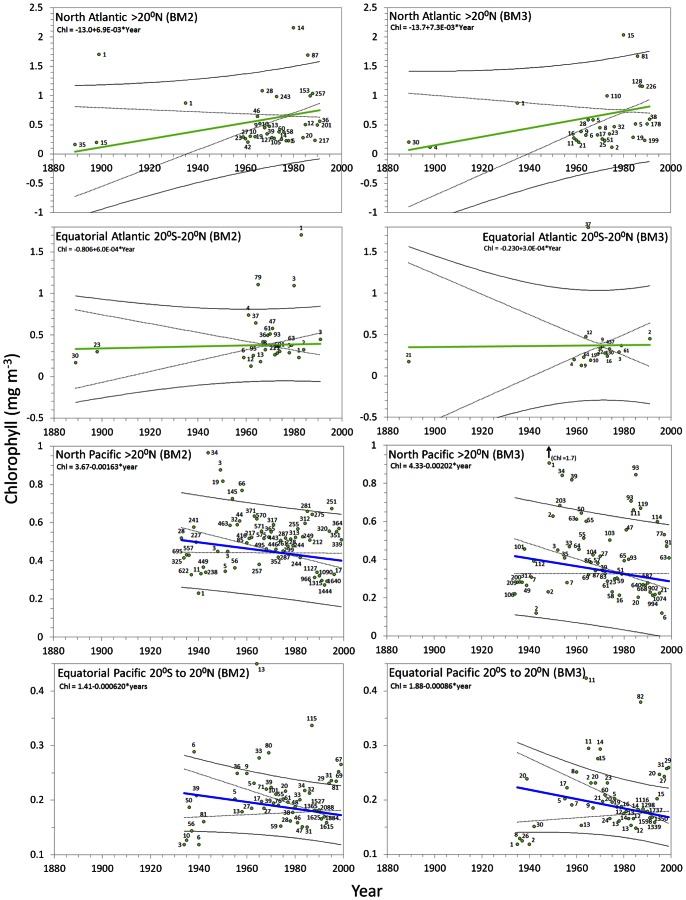
Ocean trends: The arithmetic mean derived chlorophyll concentration per year (with the no. of obs.) with superposed lines: weighted (no. of obs.) least-squares regression lines (blue or green line) indicate a bluing trend or greening trend of a sea, the 95% confidence interval of the mean (dotted line) and of the observations (solid line). A sea with no significant trend is indicated by a black line. Regression coefficients are indicated at the top of each graph.

**Figure 14:**
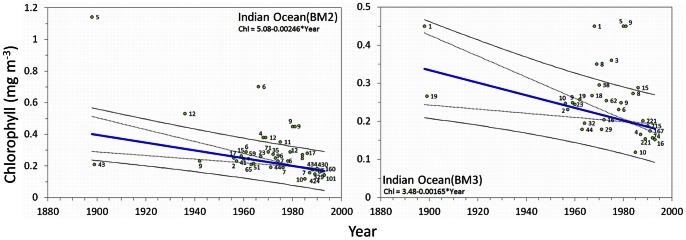
Ocean trends: the arithmetic mean derived chlorophyll concentration per year (with the no. of obs.) with superposed lines; weighted (no. of obs.) least-squares regression lines (blue or green line) indicate a bluing trend or greening trend of a sea, the 95% confidence interval of the mean (dotted line) and of the observations (solid line). A sea with no significant trend is indicated by a black line. Regression coefficients are indicated at the top of each graph.

In Figure 13 the results of the statistical analysis are presented for the Atlantic and Pacific Ocean and in Figure 14 for the Indian Ocean, per year between 1889 and 1999. The 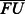 has been converted to chlorophyll according to Equation 1. For all oceans analysed, the yearly 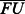 values, including the number of observations, regression coefficients and trend lines with the 95% confidence interval curves for the mean and for the observations, are presented in the graphs. At the left-hand side the graphs are based on data extracted under the BM2, graphs on the right are based on data extracted under BM3. The number of observations is indicated near the yearly means. The trend line with regression coefficients, where y is the chlorophyll concentration in mg m^−3^ and x is the year is indicated in each graph. For the oceans Table 4 shows the regression coefficient *a* with standard deviation delta *a*, Kendall's tau coefficient (represents the degree of concordance between two columns of ranked data) and the Mann-Kendall derived p-value. The last two columns of the table shows the period over which observations were collected and the start and end value of the calculated chlorophyll concentration (mg m^−3^).

**Table 4:** Results of the weighted linear regression modelling and the Mann-Kendall trend test for the oceans.

Oceans	Trend	*a*	delta	Kendall's	p-value	Sen's	Period	Chl	
			*a*	tau	(Two-tailed)	slope		start	end
***NA >20 BM2***	G	0.00692	0.0010	−0.88	<0.0001	−0.0008	1889–1991	0.04	0.75
***NA >20 BM3***	G	0.00731	0.0010	−0.05	0.022	0	1889–1991	0.07	0.82
***EQA BM2***	G	0.00060	0.0004	−0.19	<0.0001	0	1889–1991	0.33	0.39
***EQA BM3***	G	0.00031	0.0010	−0.83	<0.0001	−0.0006	1889–1991	0.35	0.38
***NP >20 BM2***	B	−0.00163	0.0001	−0.13	<0.0001	0.0001	1933–1999	0.51	0.40
***NP >20 BM3***	B	−0.00202	0.0002	−0.87	<0.0001	−0.0001	1934–1999	0.42	0.29
***EQP BM2***	B	−0.00062	0.0001	−0.08	<0.0001	0.0002	1934–1999	0.21	0.17
***EQP BM3***	B	−0.00085	0.0001	−0.76	<0.0001	0	1935–1999	0.22	0.17
***INO BM2***	B	−0.00246	0.0001	−0.25	<0.0001	0	1898–1993	0.40	0.17
***INO BM3***	B	−0.00165	0.0002	−0.21	<0.0001	0	1898–1993	0.34	0.18

Trends are indicated by a B (bluing) or a G (greening). Preceding columns show the regression coefficient *a* with delta *a*, Kendall's tau, the two-tailed p-value (with a significance level of α = 0.05) The columns with the heading Chl show the calculated start and end chlorophyll value in mg m^−3^ over the span of the trend as indicated in the column named Period (see Figures 13 and 14).

The Northern and Equatorial Atlantic show a similar greening trend for both BM2 and BM3. In the late 19th century the mean chlorophyll concentration at the surface of the North Atlantic amounts to 0.06 mg m^−3^, whereas the last year of observations shows a value of 0.79 mg m^−3^. The overall trend shows an average growth (BM2, BM3) of 0.0071 mg m^−3^ per year. The Equatorial Atlantic shows a smaller averaged chlorophyll growth of 0.0005 mg m^−3^ per year.

For the North Pacific we found a bluing trend for both BM2 and BM3 with an average decrease of −0.0018 mg m^−3^ per year. The chlorophyll concentration of around 0.47 mg m^−3^ in 1933 declined to an average of 0.35 mg m^−3^ in 1999. For the Equatorial Pacific for both BM2 and BM3, a bluing trend (on average −.00074 mg m^−3^ per year) was established between 1934 and 1999.

The results for the Indian Ocean, presented in Figure 14, show a bluing ocean for both BM2 and BM3 data with an average decay of −0.0021 mg m^−3^ per year. Between 1898 and 1993 the Indian Ocean showed significant decline in chlorophyll, from 0.37 to 0.18 mg m^−3^, at the end of the observational period.

#### Seas

**Figure 15:**
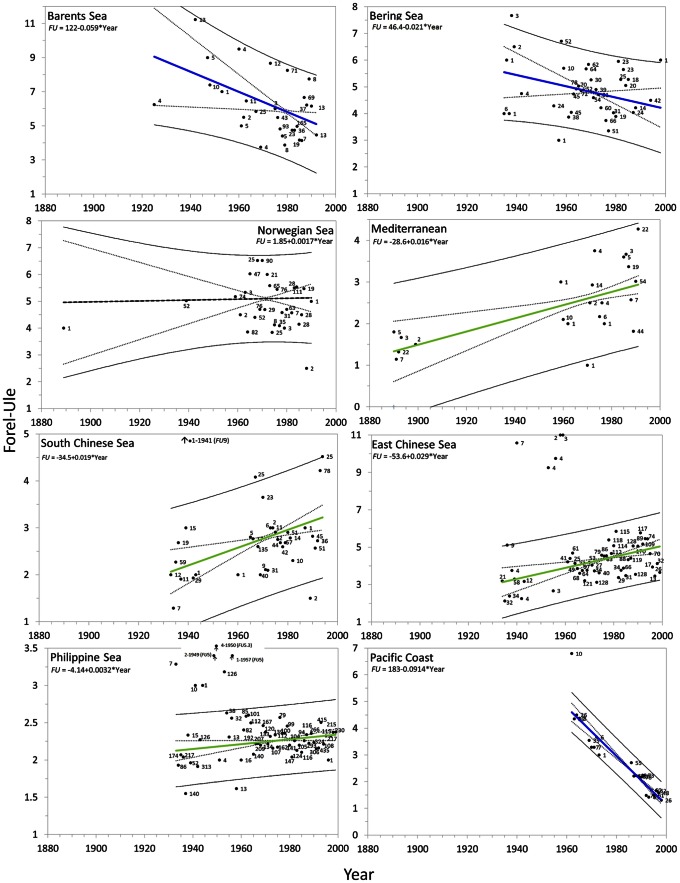
Sea trends: the arithmetic mean *FU* values (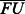) per year (with the no. of obs.) with superposed lines; weighted (no. of obs.) least-squares regression lines (blue or green line) indicate a bluing trend or greening trend of a sea, the 95% confidence interval of the mean (dotted line) and of the observations (solid line). A sea with no significant trend is indicated by a black line. Regression coefficients are indicated at the top of each graph.

**Figure 16:**
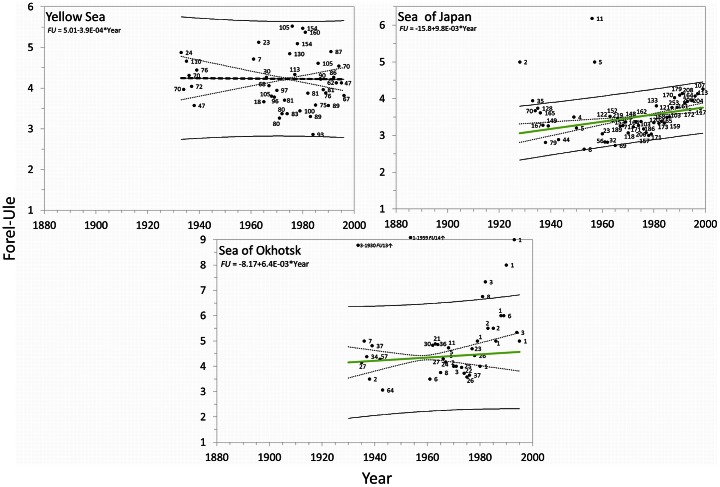
Sea trends: the arithmetic mean *FU* values (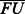) per year (with the no. of obs.) with superposed lines; weighted (no. of obs.) least-squares regression lines (blue or green line) indicate a bluing trend or greening trend of a sea, the 95% confidence interval of the mean (dotted line) and of the observations (solid line). A sea with no significant trend is indicated by a black line. Regression coefficients are indicated at the top of each graph.

For all seas analysed, the yearly 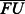 values, including the number of observations, regression coefficients and trend lines with the 95% confidence interval curves for the mean and for the observations, are presented in Figure 15 and Figure 16. Table 5 shows the general trend of a sea, bluing or greening, the weighted (no. of observations) linear regression coefficient *a* with standard deviation delta *a*, Kendall's tau and the two-tailed p-value for the analysed seas. The last two columns of the table shows the period over which observations were collected and the start and end value of the *FU* number. BM2 was used as data extraction mask. The 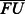 values are not converted into chlorophyll concentrations in waters with a CDOM index Φ>2.5 (Figure 12), which occurs in most of the seas. In these seas a change in CDOM as well as chlorophyll can cause shifts in sea colour.

**Table 5:** Results of the weighted linear regression modelling and the Mann-Kendall trend test for the seas.

Seas	Trend	*a*	delta	Kendall's	p-value	Sen's	Period	*FU*	
			*a*	tau	(Two-tailed)	slope		start	end
***BAR***	B	−0.0589	0.0100	−0.056	0.036	0	1925–1992	9.0	5.1
***BS***	B	−0.0211	0.0044	−0.138	<0.0001	0	1935–1998	5.2	4.6
***NWS***	-	0.0016	0.0040	0.023	0.302	0	1889–1990	5.0	5.1
***MED***	G	0.0159	0.0025	0.419	<0.0001	0.0070	1890–1991	1.3	2.9
***SCS***	G	0.0189	0.0020	0.231	<0.0001	0	1933–1994	2.1	3.2
***ECS***	G	0.0293	0.0020	0.103	<0.0001	0.0010	1934–1999	3.1	5.0
***PHS***	G	0.0032	0.0003	0.055	<0.0001	0.0003	1933–1999	2.1	2.3
***PC***	B	−0.0909	0.0020	−0.458	<0.0001	−0.0021	1962–1998	4.6	1.3
***YS***	-	−0.0004	0.0010	−0.027	0.032	0.0006	1933–1996	4.2	4.2
***SJP***	G	0.0098	0.0004	0.171	<0.0001	0.0004	1933–1999	3.1	3.8
***SOK***	G	0.0064	0.0050	0.026	0.404	0	1935–1995	4.2	4.6

Trends are indicated by a B (bluing) or a G (greening). Preceding columns show the regression coefficient *a*, with delta *a*, Kendall's tau, the two-tailed p-value (with a significance level of α = 0.05). The columns with the heading *FU* show the calculated start and end *FU* value over the span of the trend as indicated in the column named Period (see Figures 15 and 16).

Figure 15 shows for both the northern Barents and Bering Sea a bluing trend. The Barents Sea, the greenest open sea of our planet, shows a dramatic bluing (p = 0.036) with a factor of −0.059 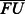 per year from ≈ 9.0 (1925) to ≈5.1 (1992). Between 1935 and 1998 the Bering Sea is bluing (p<0.0001) with a factor of −0.0211 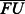 per year from ≈5.2 to ≈4.6. The Norwegian Sea, geographically situated below the Barents Sea, has an average 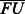 value of 5.05 and shows no significant colour change (p = 0.302) over the period 1889–1990.

The Mediterranean was geographically split into a western and eastern part, based on the known existence of an east-west oligotrophy gradient at 15° East [65], [66], [67]. In total 824 *FU* observations (BM1) between 1889 and 1999 were collected. From these only 237 observations (BM2) were analysed. Our analyses shows that the western part is slightly greener compared to the eastern part; 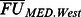 =  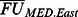
+0.8*FU*. BM1 and BM2 related 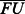 both show a significant greening between 1890 and 1991. The results for the BM2 dataset are plotted in Figure 15: the whole Mediterranean has been greening significantly over the past century, with an increase of 0.016*FU* per year since 1890. (p<0.0001). Interestingly, this greening rate is almost similar to the results found for the North Atlantic (BM3) with an increase of 0.02*FU* per year. As an indication of the potentially related changes in biomass, the *FU* data of the Mediterranean were converted into chlorophyll, assuming its water being Case1 (see Table 6). With a 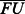 = 1.3 during 1890 and a 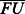 = 2.9 in the last year of observation 1991 Mediterranean chlorophyll increased from 0.13 to 0.5 mg m^−3^.

**Table 6:** The Mediterranean weighted linear regression with modelled chlorophyll, assuming an oligotrophic (case 1) classification.

Period 1890–1991	start	end
*FU*_MED_ = −28.6+0.0159 * Year	1.3	2.9
Chl_MED_ = −6.52+0.00352 * Year	0.13 mg m ^−3^	0.49 mg m ^−3^

Chlorophyll could be slightly overestimated due to the presence of CDOM. Chlorophyll values for the Mediterranean increased from the first to last decade by a factor of nearly 4.

Both the South and East Chinese Sea show greening trends (p<0.0001 for both) between 1933 and 1999; the East Chinese Sea, being greener, even shows an increase of almost two *FU* scale numbers. The Philippine Sea shows minimal greening for the period 1933 (*FU* = 2.1) −99 (*FU* = 2.3), bluing with 0.0032*FU* per year. Anomalies are identified in the periods 1940–49 and 1950–59; however, 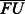 values observed during these years are based upon a limited number of observations compared to the rest of the periods.

Data of the Pacific Coast are data of open sea although the name of the area implies something else. No other sea investigated by us showed such a major change in colour, from 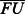 = 4.6 to 1.3 over the relatively short period 1962–98. This sea is bluing, with a rate of −0.09*FU* per year (p = <0.0001). The results of the remaining seas (Yellow Sea, Sea of Japan and the Sea of Okhotsk) are shown in Figure 16 and can be found at the end of Table 5. The central Yellow Sea (p = 0.0316) is not as yellow as its name would suggest. No significant colour change could be detected; the first observations were collected between 1933 and 1939 and have a 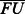 = 4.2, the same value still holds, after 60 years, for the last observational year 1996.

For the Sea of Japan an overall greening is found between 1933 and 1999 (p<0.0001). The Sea of Okhotsk, a bluish-green sea, connected with the Sea of Japan, shows an overall greening between 1935 and 1995 with an undulating pattern in 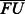 not seen so pronounced in other seas (minor trend, p = 0.4).

We emphasise that the trend analysis presented here is only a simplified description of the data. It was already shown in a previous paper on *FU* measurements made in the North Pacific [33] that oceans can show a much more complex temporal behaviour.

### Summarising the results

Lowest open ocean (>500 km off-coast) chlorophyll values of ≈0.1 mg m^−3^ were found in the North Atlantic between 1889 and1899. The North Atlantic chlorophyll shows on average a growth of 0.071 mg m^−3^ per year. The Equatorial Atlantic greens less dramatically with an amount of 0.0006 mg m^−3^ per year. Both the North- and Equatorial Pacific show a decrease in chlorophyll between 1933 and 1999, with the Equatorial Pacific bluing at about half the rate of the North Pacific. The Indian Ocean shows a continuing decline in chlorophyll over the period 1898 and 1993. Bluing seas are the Barents Sea and the Bering Sea. The Pacific Coast shows an extreme and fast bluing over a relative short period between 1962 and 1998. Like the Atlantic, the Mediterranean shows a greening trend over the analysed period. At the other side of the globe similar greening trends are found for the East- and South Chinese Sea, and, albeit less extreme, the Philippine Sea and the Sea of Okhotsk. The Sea of Japan shows a distinct and continued greening over the period 1933 to 1999. The Norwegian Sea and the Yellow Sea are, in terms of long-term colour changes, less affected seas.

## Discussion and Conclusions

We conclude that the ocean colour dataset of Forel-Ule scale observations that has been established over the period 1889 to 1999 offers a unique opportunity to investigate worldwide temporal changes of colour at the sea and ocean surface in the 20^th^ century, from 1889 to present; long-term colour changes that we show can safely be related to concentrations of chlorophyll, the most used phytoplankton biomass proxy. Bio-optical modelling, based on the state-of-the-art radiation transfer code called ‘EcoLight’ that is based on a two-component and four-component model has demonstrated how the concentration of chlorophyll and CDOM relates to ocean colour (*FU* scale index). However, we must keep in mind that the modelled chlorophyll behaves exponentially. This means that in case a *FU* observation was done over an oceanic plankton bloom (*FU*5-*FU*7, personally observed), the model generates for a *FU*5 observation 1.7 mg m^−3^ of chlorophyll and for a *FU*7 observation 6.5 mg m^−3^ of chlorophyll. Indeed, for the clearest oceanic waters (*FU* = 1 to 4), where algal pigments are the dominant factor of light absorption, a highly significant exponential relation was found between the *FU* index and chlorophyll concentration (Figure 8A). Thus, significant changes in the average chlorophyll concentration per year could be reconstructed (Figures 13 and 14). Although, for water closer to land (<200 km off-coast) the *FU*-Chlorophyll relation can be improved by a better estimate of the CDOM index and specific inherent absorption by phytoplankton in each ocean, the observed variation in colour is real and indicates a change in phytoplankton biomass at the surface.

The Forel-Ule classification of natural waters, that was started long ago, in the late nineteenth century, can, as we have shown, be regarded as an oceanographic record as reliable for interpretation as the long-term records of salinity, temperature and water transparency that are often used to relate ocean water properties to long-term climate changes that receive so much attention since the IPCC reporting on this phenomenon. The ocean colour record of the *FU*-scale observations can hardly be replaced by other data if only because it goes back all the way to 1889. We have shown here that observations made with this colour scale are not simply ‘subjective’, as earlier has been stated when Secchi disc visibility records were evaluated [68].

The Forel-Ule classification accuracy is highlighted by our analysis of the North Atlantic dataset; the annual cycle of Forel-Ule ocean colour and derived chlorophyll results (Figure 9b) are fully in line with the annual cycles established by satellite ocean colour sensors [60]. Also for the Northern Pacific the *FU*-based seasonal changes of calculated chlorophyll concentration compare well with the values of chlorophyll changes reported for this region [33] (Wernand and van der Woerd, 2010b).

We are well aware of the fact that in some seas the number of observations available and used for our analyses is rather low, but they are at the same time the only data related to phytoplankton biomass change in these ocean regions. The Mann-Kendall analysis was performed to evaluate the ocean colour or chlorophyll trend of an ocean or sea. The outcome of the analysis indicates that the North Atlantic and Equatorial Atlantic have been greening over the whole past century. The increase in North Atlantic chlorophyll after the 1950's is consistent with the trend of increase in the Phytoplankton Colour Index (PCI) obtained during the Continuous Plankton Recorder (CPR) survey over the past 50 years [14] [69], [70], [71], [72]. Further evidence of a greening Atlantic is given by Edwards *et al*. [73] in a ‘SAHFOS ecological status report’ of 2007/2008 (‘*there has been a large increase in phytoplankton since the late 1980s in most regional area of the North Atlantic’*). For the five oceans under investigation a significant trend was found (p<0.0001, significance level of 0.05). Out of eleven investigated seas seven show trends with a p<0.0001.

Our findings do not indicate a *global* trend in the ocean's colour as established on the basis of the *FU* record, and therewith in its chlorophyll contents. We cannot confirm the recent report of an ocean-wide phytoplankton decrease [23]; Boyce *et al*. found a global decline of phytoplankton over the past century that they related to increasing sea surface temperature. Differences in data filtering techniques may have been the cause of the considerable discrepancy between the results of Boyce *et al*. [23] and the results we present here. Boyce *et al*. based their conclusions on datasets from which only data obtained in waters <25 m deep or <1 km from the coast were excluded. This means that their results close to coasts may have been biased by chlorophyll-rich eutrophic coastal zones. This will have an impact on trends in oligotrophic regions, where chlorophyll varies much less. In contrast to Boyce *et al.* [23], we have omitted data <100 km (seas and oceans) or <500 km (oceans) from the coast.

Henson *et al*. [74] recently concluded that detection of climate change-driven trends in the satellite data record is confounded by the relatively short time series and large inter-annual and decadal variability in productivity. According to Henson *et al.*, recently observed changes in chlorophyll, primary production and the size of the oligotrophic gyres cannot be explicitly ascribed to the impact of global climate change. They suggest that time series of at least 40 years are needed to distinguish trends from natural variability.

Gregg and Conkright [60] re-analysed the CZCS global ocean chlorophyll product, using SeaWiFS compatible atmospheric correction methods, and made a first quantitative comparison of the decadal trends in global ocean chlorophyll over the periods 1979–1986 (CZCS) and 1997–2000 (SeaWiFS). They reported a decrease in global chlorophyll concentrations from the CZCS records to present of about 6%; they found larger reductions in the northern high latitudes and an increase in chlorophyll in the low latitudes. Their conclusions are not in line with ours but we must bear in mind that results are based upon the results of two relatively short periods – much shorter than the shortest period presented by us. Antoine *et al*. [19] also used CZCS and SeaWiFS data; they reported an overall increase of the world ocean average chlorophyll concentration by about 22%.

## Recommendations

The dataset presented here contains 220,000 Forel-Ule observations; 160,000 remain unexploited for the time being. It would be of great interest to investigate this latter dataset, collected in coastal and shelf seas (<100 km off-shore) globe-wide, to establish the possible influence of coloured near shore waters on open-ocean colour; moreover, with a refinement of the model results presented here it should be possible to classify water quality not only in the open ocean but also in seas where coloured dissolved organic matter (CDOM) is not co-varying with chlorophyll, usually sea regions where human influence plays a role, notably eutrophication by nutrient input of rivers, and by sediment and CDOM load changes. However, we must bear in mind that such a refinement is not an easy task as chlorophyll and CDOM absorption can have a similar influence on sea and ocean colour and therefore on *FU* values. Future work should concentrate on explanations of driving forces behind the decadal trends in open-ocean colour presented here, and anomalies in the ocean colour record over long terms of up to a century. Hindcasting is the only way to understand and predict changes in the ocean's ecosystem in its relation to climate change.

Long-term series are limited; a literature search indicates that ‘long-term’ generally means: between 5 and at most 30 years. It is therefore not only challenging but also necessary to combine archived long-term data series of basic water quality parameters such as Secchi disc visibility depth, chlorophyll, or phytoplankton abundance (e.g., the colour index recorded since 1948 during the Continuous Plankton Recorder Survey of SAHFOS) to establish inter-annual or inter-decadal means from which results can be compared to our results. An attempt to combine historic water quality datasets has been presented recently by Boyce et *al.* [75] (2012). Data of other long-term time series, namely the Hawaii- and Bermuda Atlantic Ocean Time-Series (HOTS and BATS, both since 1988) and the California Cooperative Oceanic Fisheries Investigations (CalCOFI since 1949) should help to achieve this goal.

Finally, we like to make a plea, substantiated in the present paper, for the reintroduction of the *FU*-scale to classify the colour of seas and oceans. Ocean colour classification by means of a single numerical value instead of a hyper-spectral classification will facilitate the interpretation of long-term ocean colour data series and at the same time facilitate a connection between the present and the past. MERIS satellite Forel-Ule mapping can play a role here, through validation and coupling of historic observations to the present era of satellite-based monitoring.
